# Burnout Levels in Nurses and Associated Factors during the COVID-19 Pandemic—A Cross-Sectional Study

**DOI:** 10.3390/healthcare11142032

**Published:** 2023-07-16

**Authors:** Karolina Filipska-Blejder, Anna Antczak-Komoterska, Magdalena Kostecka, Beata Haor, Agnieszka Królikowska, Renata Jabłońska, Lech Grzelak, Mariusz Wysokiński, Wiesław Fidecki, Adam Wiśniewski, Robert Ślusarz

**Affiliations:** 1 Neurological and Neurosurgical Nursing Department, Faculty of Health Science, Collegium Medicum in Bydgoszcz, Nicolaus Copernicus University in Toruń, 85-821 Bydgoszcz, Poland; agakrolikowska6@wp.pl (A.K.); renjab_1@wp.pl (R.J.); robert_slu_cmumk@wp.pl (R.Ś.); 2 Faculty of Health Sciences, State University of Applied Sciences in Wloclawek, 87-800 Wloclawek, Poland; antczak231@wp.pl (A.A.-K.); kina14szkola@wp.pl (M.K.); beata.haor@interia.pl (B.H.); lechg7@gmail.com (L.G.); 3 Department of Fundamentals of Nursing, Chair of Nursing Development, Faculty of Health Sciences, Medical University of Lublin, 20-093 Lublin, Poland; mariusz.wysokinski@umlub.pl (M.W.); fidecki@interia.pl (W.F.); 4 Department of Neurology, Faculty of Medicine, Collegium Medicum in Bydgoszcz, Nicolaus Copernicus University in Toruń, 85-094 Bydgoszcz, Poland; adam.lek@wp.pl

**Keywords:** burnout, COVID-19, nurses, occupational burnout syndrome

## Abstract

Previous studies have shown that sudden changes in the nature of nursing work and their work environment related to the COVID-19 pandemic have affected the professional experience of nurses, and consequently led to an increase in professional burnout in this professional group. Thus, the aim of the study was to measure occupational burnout among nurses working during the COVID-19 pandemic in Poland. A cross-sectional study was conducted with pediatric and surgery female nurses (N = 110, mean age 51 ± 6.92) from the Provincial Specialist Hospital in Włocławek, Poland. The participants completed the Link Burnout Questionnaire (LBQ) and the Socio-Demographic Questionnaire (SDQ). The data were analyzed using Spearman’s rank correlation and Mann–Whitney U test. The study showed that high burnout affected 6.4% of nurses. The level of professional burnout for the subscales of psychophysical exhaustion, relationship deterioration, professional inefficacy and disappointment was 28.2%, 26.4%, 11.8% and 13%, respectively (mean score: 19.85 ± 6.51, 18.03 ± 5.15, 13.74 ± 4.07 and 17.61 ± 5.85, respectively). The results show that surgical nurses were statistically more likely to experience professional burnout. In sum, burnout among nurses has become a serious problem, especially considering the COVID-19 pandemic, which is why it is so important to continue research in this area. Hospital management needs to take urgent action to address the systemic and professional issues that contribute to the suboptimal mental health of nurses.

## 1. Introduction

Occupational burnout has been recognized as a serious and growing occupational hazard in the international arena for several decades [1,2]. Due to the seriousness of the situation, in 2019, the World Health Organization included burnout in the 11th Revision of the International Classification of Diseases (ICD-11) as an occupational phenomenon. ICD-11 defines burnout as follows: “Burn-out is a syndrome conceptualized as resulting from chronic workplace stress that has not been successfully managed. It is characterized by three dimensions: feelings of energy depletion or exhaustion; increased mental distance from one’s job, or feelings of negativism or cynicism related to one’s job; and reduced professional efficacy. Burn-out refers specifically to phenomena in the occupational context and should not be applied to describe experiences in other areas of life.” [3].

The contemporary problem of occupational burnout is growing, which is mainly due to the importance that is currently attributed to one’s professional career and job characteristics. The causes of occupational burnout can be divided into those caused by working conditions and those resulting from the individual characteristics and character of a given person. Occupational burnout is particularly common among nurses and people from the healthcare sector, because their work is inextricably linked to stress, excess duties, shift work, a sense of responsibility, showing empathy and commitment, in addition to the need to maintain emotional distance. First of all, it is a job that requires establishing close interpersonal contacts, often with a sick and suffering person fighting for their health and life. In addition, the ongoing COVID-19 pandemic has caused anxiety and fear among medical staff, which in turn may lead to an even greater increase in burnout rates [4,5]. Many ongoing scientific projects also emphasize the significant impact of shift work. Studies conducted by Jaradat et al. [6] indicate that nurses working in shifts had a much higher level of mental stress than people who worked day shifts.

Over the past few years, professional burnout among nurses has become a serious problem around the world, especially considering the COVID-19 pandemic. It has been shown that mental health, work-related stress and work–life interference closely correlate with occupational burnout among nurses. However, the basic mechanisms behind this correlation have not yet been fully explored and understood; therefore, further research in this area is still necessary [5]. In addition, there are large discrepancies in the published results regarding the prevalence of occupational burnout among nurses. Woo et al. [7] published a systematic review and meta-analysis on the prevalence of burnout among over 40,000 nurses of various specialties from 49 countries. It was shown that global indicators of burnout symptoms were observed in approx. 11% of nurses. There were significant differences in the measurement of occupational burnout depending on the specialization, geographical region and the measurement tool used. Ślusarz et al. [8], in their review, also point to high prevalence rates of occupational burnout and large discrepancies in the obtained results ranging from 14.3% to 84.7%. The highest burnout rate was observed among nurses who worked in the ICU during the COVID-19 pandemic. Almeida and Poeira [9] assessed burnout in nurses in an Intensive Care Unit during COVID-19. Fairly high burnout rates have been observed with greater expression in the emotional exhaustion and personal fulfillment domains. Occupational and sociodemographic variables did not have a statistically significant effect on any of the three dimensions of burnout. Sagherian et al. [10] assessed the severity of insomnia, fatigue and the psychosocial well-being of 587 hospital nurses and nursing assistants. Moderate-to-high chronic fatigue, subthreshold insomnia, high acute fatigue and low-to-moderate intershift recovery were reported. In addition, the highest burnout rates were observed in the aspects of emotional exhaustion, depersonalization and increased personal accomplishment. Furthermore, those caring for COVID-19 patients scored significantly worse in all measurement categories.

Nurse burnout was an important public health issue even before the outbreak of the pandemic. Moreover, studies have observed that ongoing viral epidemics, including COVID-19, have a negative impact and exacerbate the problem [11,12,13,14,15]. Professional burnout is a response to chronic fatigue and stressors at work. It was recognized as a serious and widespread occupational hazard during the COVID-19 pandemic. Thus, the aim of our study was to analyze and characterize the phenomenon of occupational burnout among nurses employed in pediatric and surgical wards.

## 2. Materials and Methods

### 2.1. Participants and Settings

From November 2020 to July 2022, we conducted this cross-sectional study in the Department of Pediatrics and Surgical Department at the Provincial Specialist Hospital in Włocławek, Poland. The study included people who met the inclusion criteria: actively employed in the Pediatric or Surgical Department; working a full-time job; having at least one year of experience; voluntarily agreed to participate. Of the whole sample of nurses (N = 110), 100% were female, with an average age of 51 years; 60.9% worked in the Surgical Department; 34.5% had 31–35 years of job experience; and 35.5% had 31–40 years of experience in the current department. Table 1 shows the participants’ sociodemographic characteristics.

The anonymous survey project allowed for the collection of reliable and credible data. When completing the questionnaire, the nurses could always ask a question to the researcher who was present nearby. The questions concerned the last 3 months. In the beginning, the researchers provided each participant with information about the subject and purpose of the project and the rules applicable during its duration. Participation in the project was voluntary; nurses were guaranteed anonymity and confidentiality. Informed consent was obtained from each subject.

### 2.2. Variables and Measurements

The Link Burnout Questionnaire (LBQ) [16,17] and the Socio-Demographic Questionnaire (SDQ) were used in the presented project. The LBQ is designed to measure occupational burnout in people working in professions related to helping other people and teaching using four subscales (six items each): (1) psychophysical exhaustion (the dimension concerning the assessment of one’s own psychophysical resources, questions 1, 7, 10, 16, 22, 24); (2) deterioration of relationships with users (dimension describing the quality of relationships with patients, questions 3, 5, 12, 14, 18, 23); (3) job ineffectiveness (dimension referring to the assessment of one’s own professional competences, questions 2, 8, 11, 13, 17, 20); and (4) disappointment (dimension of existential expectations, questions 4, 6, 9, 15, 19, 21). The method of providing answers is based on a 6-point Likert scale, the individual points of which correspond to the following categories: never—1 point; rarely—2 points; once or more times a month—3 points; more or less every week—4 points; several times a week—5 points; and every day—6 points. The raw score on each scale is calculated by summing up the scores for each item. The original coefficient alpha was 0.77 for psychophysical exhaustion, 0.69 for deterioration of relations with users, 0.68 for job ineffectiveness and 0.85 for disappointment [17]. The SDQ contained questions on age, gender, place of residence, education (diploma in nursing, bachelor of nursing, master’s degree in nursing), years of experience in current department, overall years of experience and current area of work (Department of Pediatrics or Surgical Department).

### 2.3. Ethical Statement

The study was approved by the Bioethics Committee of the State Vocational University in Włocławek (approval no. 14/20) and Director of the Provincial Specialist Hospital in Włocławek, Poland. The study was conducted according to the Declaration of Helsinki regarding research on humans. All subjects provided informed consent to participate in the study. 

### 2.4. Statistical Analysis

The statistical analysis was performed with STATISTICA version 13.1 (Dell Technologies, Round Rock, TX, USA). Mean, standard deviation, frequency and percentage were used for the presentation of the descriptive data. The following tests were performed: Spearman’s rank correlation (internal correlations of LBQ scales; correlations of age groups, education, seniority); and Mann–Whitney U test (differences in LBQ scales between hospital departments, place of residence). A *p*-level < 0.05 was considered statistically significant.

## 3. Results

The average point value of the subscale of psychophysical exhaustion was 19.95 points. Generally, 28.2% of nurses were characterized by a high level of professional burnout in terms of psychophysical exhaustion. Of all the items of psycho-physical exhaustion, the highest rated item was the following: I feel tense at work (M = 3.0, SD = 1.30). The worst was as follows: I feel that my work exhausts me physically (M = 3.72, SD = 1.47). The average score for not engaging in relationships was 18.03 points. Only 4.5% were engaged people who did not have the problem of a lack of commitment in relationships. Another 26.4% were people with a high level of professional burnout in terms of a lack of commitment to relationships. Out of all the aspects relating to a lack of commitment to relationships, the following was rated as the highest: relationships with my clients (subordinates, patients) give me satisfaction (M = 2.67, SD = 1.29). The worst was as follows: I think I deal with more difficult clients (subordinates, patients) than others (M = 3.51, SD = 1.54). The average score for professional ineffectiveness was 13.74 points. Nearly 4% of nurses were effective workers without feeling professionally ineffective. Another 11.8% were people with a high level of professional burnout in terms of a sense of professional ineffectiveness. Out of all the aspects related to a sense of professional ineffectiveness, the highest rating was given to the following: I feel that I am able to properly organize the work required for my position (M = 1.65, SD = 0.80). The worst was as follows: I feel that I am unable to face the problems of my clients (subordinates, patients) (M = 2.88, SD = 1.01). The average point value of the disappointment subscale was 17.61 points. Only 8% of the respondents were people who did not have problems with disappointment or feeling satisfied. On the other hand, nearly 13% were people with a high level of professional burnout in terms of disappointment. Of all the aspects related to disappointment, the highest-rated aspect was as follows: I doubt that what I do has any value (M = 2.18, SD = 1.21); the lowest was as follows: I feel that my professional ideals still motivate me to work (M = 3.43, SD = 1.42). Moreover, internal statistically significant correlations of LBQ scales were found at the medium level (*p* < 0.05) (Table 2). 

The subjects working in the pediatric ward obtained better results in all LBQ subscales. Using the Mann–Whitney test, a statistically significant difference between the departments was observed regarding the results of the subscale psychophysical exhaustion (*p* = 0.020), while the result of the subscale sense of professional ineffectiveness was borderline significant (*p* = 0.085). It was also shown that people aged 56–61 obtained the best results in the subscale of psychophysical exhaustion (M = 17.59, SD = 6.24), relationship deterioration (M = 17.21, SD = 5.41) and disappointment (M = 16.62, SD = 6.54). On the other hand, nurses aged 51–55 were characterized by a high level of occupational burnout in terms of psychophysical exhaustion (M = 22.06, SD = 6.47), professional inefficacy (M = 14.44, SD = 4.63) and disappointment (M = 18.53, SD = 5.47). It should also be emphasized that rural women obtained better results in all subscales except for disappointment. People with a master’s degree had the highest level of occupational burnout in terms of relationship deterioration (M = 18.89, SD = 4.81) and professional inefficacy (M = 14.11, SD = 3.91). On the other hand, people with the least work experience in the current ward (11–20 years) turned out to be the most professionally burnt-out in all dimensions of the LBQ scale (Table 3).

## 4. Discussion

In this study we conducted on the work environment of pediatric and treatment nurses during the COVID-19 pandemic, there was a phenomenon of professional burnout at an average level; 6.4% of nurses had a high level of professional burnout. An earlier study found that the rate of burnout among nurses varied across studies. Gravante et al. [18] assessed the incidence of burnout and the quality of life among Italian nurses during the COVID-19 pandemic. Emotional exhaustion appeared in 52.3% of cases and depersonalization was observed in 85%. Galanis et al. [19], in their systematic review and meta-analysis, assessed nurse burnout and related risk factors during the COVID-19 pandemic. Of the sixteen studies involving 18,935 nurses, the overall incidence of depersonalization was 12.6%, and that of emotional exhaustion was 34.1%. In turn, Karadağ and Çiçek [20] determined that nearly 53% of nurses experienced professional burnout, and about 27% needed professional help. Izdebski et al. [21] assessed the level of occupational burnout among 2196 Polish healthcare professionals who worked with patients during the COVID-19 pandemic. The prevalence of burnout ranged from 27.7% among other non-medical workers to 36.5% among nurses.

Nurses, as healthcare professionals, play a crucial role in the treatment and prevention process, and most importantly, they provide direct patient care. Work-related stress factors such as fatigue, sleep disturbances and burnout negatively impact quality of care, quality of life, clinical decision making, practice and clinical competence [22]. Undoubtedly, the COVID-19 pandemic exacerbated the problem of professional burnout among healthcare professionals. One of the many challenges they had to face was undoubtedly caring for a patient with a new, unknown infectious disease. Working in new and insecure conditions, having a frequent lack of necessary personal protective equipment, working overtime, fear for one’s own health and the risk of infecting relatives are just some of the factors that have become predictors of occupational burnout [23]. Our study showed that people working in the pediatric ward experienced psychophysical exhaustion statistically less often than treatment nurses. With the general increase in the number of COVID-19 patients, the likelihood of the SARS-CoV-2 virus appearing among patients qualified for surgical procedures and admitted in emergency situations is progressively increasing. If a patient is admitted to the hospital during the incubation period of the virus, the risk of infection among the surgical ward staff itself increases. Therefore, nurses in surgical wards may have experienced anxiety and mental stress to a greater extent during the COVID-19 pandemic. In addition, we showed that people aged 51–55, residents of the city and nurses with the least work experience in a given ward most often experienced professional burnout. Galanis et al. [19] also observed that a younger age was the main risk factor increasing burnout among nurses. Stress is often found to be more prevalent in younger or less experienced individuals, and therefore preventive interventions should focus on this aspect. Furthermore, the focus should be on the effectiveness of peer interventions or peer support, from older or more experienced nurses to younger ones. Luceño-Moreno et al. [23] showed that symptoms of depression were a common prognostic variable for all dimensions of burnout. In turn, age and seniority were predictors of depersonalization. Savsar and Karayurt [24] assessed factors affecting burnout and anxiety among surgical nurses in the COVID-19 pandemic. Moderate levels of anxiety and burnout were observed. In addition, factors such as female gender and a belief in the lack and shortage of medical personnel turned out to be significant predictors of anxiety and occupational burnout. 

Kim [22] showed that education among nurses in the field of COVID-19 has a significant impact on practice. Therefore, it can be concluded that education is an important strategy for improving practice. Given the low rate of correct answers for basic knowledge such as the causes, management and mortality of COVID-19, it is essential to conduct systematic education and deepen nurses’ knowledge of basic information and guidelines, as well as to study the effectiveness of education. Furthermore, the modern human lives in an era of significant civilization development associated with an ever-increasing pace of life, increasing requirements of employers and changing labor laws and employment structures, where professional work is the most important activity in the life of every adult. In the workplace, various situations that trigger strong tensions and anxiety can occur, causing stress. Nursing is one of the professions in which stress is an inherent property and results from the very nature of the profession. The work of nurses carries special mental burdens, the source of which is another person who is often in a difficult situation [3,8]. Nurses work under strong and long-lasting emotional tension. An inability to cope with stress and a lack of support from one’s team in extreme situations leads to the development of professional burnout syndrome, which not only significantly reduces the quality of work performed and prevents nurses from their further professional development, but also has a detrimental effect on mental and physical health and the functioning of the entire healthcare system. That is why it is so important for employers and employees to take action to prevent burnout syndrome. Carrying out continuous research in order to better and thoroughly understand the phenomenon of occupational burnout and to search for effective interventions in preventing and mitigating its negative effects is thus needed [15,25,26,27,28].

This study has several limitations. First, the study design was cross-sectional. This means that it is not possible to determine the reliable causality of the factors affecting the level of occupational burnout. It is necessary to design longitudinal studies in order to accurately characterize the described phenomenon. Moreover, no such studies have been conducted in the pre-pandemic period, which prevents us from comparing our results and trends in the same study group in the pre- and post-pandemic period. On the other hand, it is worth noting that the time of data collection, the COVID-19 pandemic, may affect the results of this study. Furthermore, the data collected span more than two years. At different time points, the conditions varied, likely influencing the causes of the studied effect. The impact of COVID-19 on the findings needs to be interpreted with caution. Thirdly, the study was conducted in only one hospital, in selected wards. Ultimately, the sample size of this study was small. It is therefore necessary to continue the study and expand the group of surveyed nurses.

## 5. Conclusions

This study showed that high burnout affected 6.4% of nurses working in the Department of Pediatrics and Surgical Department at the Provincial Specialist Hospital in Włocławek during the COVID-19 pandemic. In turn, in individual subscales, the highest level of occupational burnout was as follows: 28.2% for psychophysical exhaustion, 26.4% for relationship deterioration, 11.8% for professional inefficacy and 12.8% for disappointment. It has been shown that people working in surgical wards experience burnout, in terms of psychophysical exhaustion, statistically more often. It is necessary to conduct further research and take action to improve the current working environment of nurses, regulate their working time, enable personal and professional development and offer support at each stage of their development and professional career. 

## Figures and Tables

**Table 1:** The participants’ sociodemographic characteristics.

Variable	All Participants (N = 110)			
	N	%	Mean	SD
Department of				
Pediatrics	43	39.1		
Surgical	67	60.9		
Age			51.0	6.92
≤45 years	17	15.5		
46–50 years	28	25.5		
51–55 years	36	32.7		
56–61 years	29	26.4		
Place of residence				
Village	24	21.8		
City	86	78.2		
Education				
Diploma in nursing	39	35.5		
Bachelor of nursing	44	40.0		
Master of nursing	27	24.5		
Years of experience in the current department			21.4	12.90
≤10 years	35	31.8		
11–20 years	14	12.7		
21–30 years	22	20.0		
31–40 years	39	35.5		
Overall years of experience			29.0	8.83
≤20 years	20	18.2		
21–30 years	30	27.3		
31–35 years	38	34.5		
>35 years	22	20.0		

**Table 2:** Characteristics of LBQ subscales and assessment of correlations between them using the Spearman’s rank correlation test.

Subscales of LBQ	M	SD	PE	RD	PI	DI
			R	R	R	R
Psychophysical exhaustion (PE)	19.95	6.51	---	0.632 *	0.478 *	0.622 *
Relationship deterioration (RD)	18.03	5.15	0.632 *	---	0.595 *	0.526 *
Professional inefficacy (PI)	13.74	4.07	0.478 *	0.595 *	---	0.618 *
Disappointment (DI)	17.61	5.85	0.622 *	0.526 *	0.618 *	---

*—significant dependencies; Spearman’s rank correlation test.

**Table 3:** Analyses of factors associated with occupational burnout (LBQ subscales).

Characteristic	Total Sample (N = 110)							
	Psychophysical Exhaustion		Relationship Deterioration		Professional Inefficacy		Disappointment	
	M (SD)	Statistical Result	M (SD)	Statistical Result	M (SD)	Statistical Result	M (SD)	Statistical Result
Department		Z = 2.323 *p* = 0.020 ^a^		Z = 1.057 *p* = 0.291 ^a^		Z = 1.724 *p* = 0.085 ^a^		Z = 0.562 *p* = 0.574 ^a^
Pediatrics	18.23 (6.14)		17.42 (4.57)		12.86 (3.47)		17.05 (5.22)	
Surgical	21.06 (6.55)		18.42 (5.49)		14.30 (4.34)		17.97 (6.23)	
Age		R = −0.052 *p* = 0.592 ^b^		R = −0.045 *p* = 0.638 ^b^		R = 0.027 *p* = 0.780 ^b^		R = −0.024 *p* = 0.803 ^b^
<45 years	18.12 (6.34)		19.35 (5.72)		13.29 (3.50)		17.41 (6.61)	
46–50 years	20.82 (6.15)		16.86 (4.03)		13.25 (3.43)		17.57 (5.17)	
51–55 years	22.06 (6.47)		18.97 (5.33)		14.44 (4.63)		18.53 (5.47)	
56–61 years	17.59 (6.24)		17.21 (5.41)		13.59 (4.28)		16.62 (6.54)	
Place of residence		Z = 0.211 *p* = 0.833 ^a^		Z = 0.610 *p* = 0.542 ^a^		Z = 1.269 *p* = 0.204 ^a^		Z = −0.395 *p* = 0.692 ^a^
Countryside	19.92 (6.36)		17.75 (5.78)		12.63 (3.21)		17.71 (5.15)	
City	19.97 (6.59)		18.10 (5.00)		14.05 (4.24)		17.58 (6.05)	
Education		R = −0.128 *p* = 0.181 ^b^		R = 0.090 *p* = 0.351 ^b^		R = 0.056 *p* = 0.561 ^b^		R = −0.099 *p* = 0.304 ^b^
Diploma in nursing	20.44 (6.47)		17.67 (5.11)		13.49 (3.86)		18.67 (6.29)	
Bachelor of nursing	20.50 (7.19)		17.82 (5.45)		13.73 (4.41)		16.64 (5.58)	
Master’s degree in nursing	18.37 (5.25)		18.89 (4.81)		14.11 (3.91)		17.67 (5.53)	
Years of experience in current department		R = −0.111 *p* = 0.250 ^b^		R = −0.129 *p* = 0.179 ^b^		R = −0.013 *p* = 0.894 ^b^		R = 0.067 *p* = 0.485 ^b^
≤10 years	19.31 (6.71)		18.60 (5.81)		13.31 (4.14)		16.49 (5.59)	
11–20 years	24.29 (6.84)		20.50 (5.68)		15.36 (5.18)		20.00 (5.26)	
21–30 years	20.82 (6.09)		17.68 (4.05)		14.23 (3.05)		17.77 (5.61)	
31–40 years	18.49 (5.90)		16.82 (4.67)		13.26 (4.04)		17.67 (6.32)	
Overall years of experience		R = −0.091 *p* = 0.347 ^b^		R = −0.115 *p* = 0.233 ^b^		R = 0.011 *p* = 0.911 ^b^		R = 0.033 *p* = 0.734 ^b^
≤10 years	18.10 (5.50)		19.55 (5.71)		13.70 (3.56)		17.35 (6.23)	
21–30 years	21.77 (6.52)		17.30 (4.12)		12.60 (3.16)		16.60 (4.61)	
31–35 years	21.34 (6.43)		18.68 (5.32)		15.32 (4.76)		19.34 (5.53)	
>35 years	16.77 (6.26)		16.50 (5.38)		12.59 (3.62)		16.23 (7.07)	

^a^ Mann–Whitney U test; ^b^ Spearman’s rank correlation test.

## Data Availability

The data presented in this study are available on request from the corresponding author. The data are not publicly available due to respondents’ privacy.
